# COVID-19 suspicion revealed to be fat embolism syndrome

**DOI:** 10.11604/pamj.2020.36.104.23631

**Published:** 2020-06-17

**Authors:** Salim Mazouz, Ouissal Aissaoui, Mohamed Anass Fehdi, Afak Nsiri, Rachid AlHarrar

**Affiliations:** 1 Department of Anesthesiology and Critical Care Medicine, COVID-19 Dedicated ICU, University Hospital of Casablanca, Casablanca, Morocco

**Keywords:** COVID-19, Fat embolism syndrome, acute respiratory distress

## Abstract

The novel coronavirus, named SARS-CoV-2, responsible of the COVID-19 is now causing a pandemic. Detecting all possible cases and eliminating differential diagnoses in front of any acute respiratory distress has become a daily challenge for doctors around the world. We believe that non-COVID patients are the hidden victims of the actual health problematic. We report from this manuscript the case of a patient with fat embolism syndrome that has been suspected as COVID-19.

## Introduction

The novel Coronavirus, named SARS-CoV-2, is responsible of the COVID-19. It is a viral pneumonia that appeared in December 2019 in Wuhan, China, and is now causing a pandemic. Nowadays, doctors around the world are facing a great challenge, dealing with COVID-19 patients, but also, not misdiagnosing non-COVID patients. In fact, we believe that non-COVID patients are the hidden victims of the actual health problematic. Several patients with respiratory distress are wrongly suspected as COVID-19. Fat embolism syndrome (FES) is a rare incident, in post-traumatic bone injuries. It might occur in post-operatory bone surgeries. We report the case of a patient with FES that has been suspected as COVID-19 patient.

## Patient and observation

**Figure 1:**
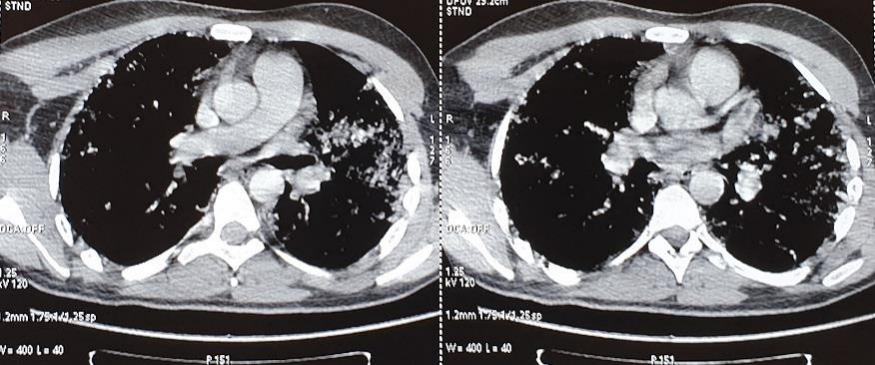
findings on computed tomography (CT) on admission showing no evidence of detectable pulmonary embolism

**Figure 2:**
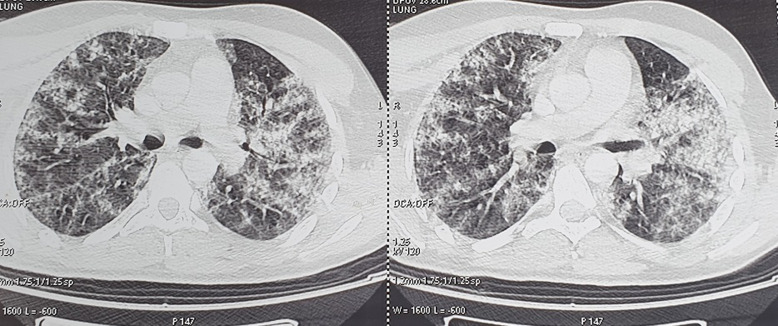
findings on chest computed tomography (CT) on admission showing multiple frosted glass areas and central and peripheral consolidation diffusely distributed over the two lungs

A 33-year-old man with no medical past history, victim of a right leg trauma resulting in a closed tibial shaft fracture initially treated on the 4^th^ of May 2020 in orthopedic surgery by a centro-medullary nailing. On the 6^th^ of May he presented an acute respiratory distress with fever (40.1°C) of which then a high-resolution chest computed tomography (CT) was performed, showing bilateral ground-glass opacities, with multilobe central and peripheral involvement (Figure 1, Figure 2). Given the current pandemic context, the patient was suspected of having a respiratory infection with SARS-CoV-2 and was transferred to the intensive care unit dedicated to the management of COVID-19.

On admission, the clinical evaluation revealed a normal neurological and hemodynamic state, the respiratory rate was at 40, the oxygen saturation at 52% without oxygen, and at 96% with high-concentration mask. Arterial blood gas analysis showed respiratory alkalosis with hypoxemia, arterial oxygen pressure (PaO_2_) at 55 mmHg. The initial conditioning included, a peripheral intravenous line (PIV), a high-concentration oxygen mask, a half-seated position (45°). Biological assessment had revealed an important inflammatory syndrome with high CRP at 277 mg/L, a hemoglobin level of 10.4 g/L, hematocrit at 30% and hypoalbuminemia at 33 g/L without lymphopenia nor hyperferritinemia. The troponin level was high at 82.1 μg/L and CPK at 375 mg/l. Reverse transcription polymerase chain reaction (RT-PCR) on nasopharyngeal swab did not detect SARS-CoV-2 RNA. We also noted a drop in total cholesterol to 1.22 g/L and a low HDL level at 0.33 g/L. The renal and hepatic function, the electrolytes and the plasma procalcitonin were moreover without particularity.

## Discussion

Novel coronavirus 2019 (COVID-19) also known as severe acute respiratory syndrome coronavirus 2 (SARS-CoV-2) is an enveloped, non-segmented positive-sense RNA virus belonging to the beta-coronaviridae family. This virus is known to cause severe bilateral pneumonia and acute respiratory distress syndrome (ARDS) which can lead to difficulty breathing requiring mechanical ventilation and intensive care unit management.

Fat embolism syndrome (FES) is an event following a traumatic injury, and its pathophysiologic mechanism continues to be elusive. It generally occurs when a bone marrow fat enters the bloodstream resulting in a cascade of inflammatory response, hyper-coagulation, and an array of symptoms that generally begin within 24 to 48 hours [1]. The detection of all possible cases of SARS-CoV-2 infection has become a global issue and has caused recently many doctors to suspect COVID-19 in the presence of acute respiratory distress.

Although typical and atypical CT image findings of COVID-19 are reported in current studies, the CT image features of COVID-19 overlap with other respiratory diseases [2], and the selection of appropriate detection techniques and methods for accurate and rapid identification of pathogens including SARS-CoV-2 RNA test, therefore plays a key role in improving the diagnosis of the patients [3]. Fat embolism syndrome is a differential diagnosis that should not be ruled out especially when faced with respiratory distress in a patient surgically treated for a tibial fracture. The radiological findings in FES are similar to those in COVID-19 [4].

## Conclusion

Differential diagnoses of SARS-CoV-2 infection are a real challenge for doctors, and a bundle of arguments makes it possible to differentiate between each.
